# New Medical Journal

**Published:** 1859-05

**Authors:** 


NEW MEDICAL JOURNAL.—We have received No. 4 of the *St. Joseph Medical Journal of Medicine and Surgery*, under the editorial management of Drs. T. H. Crane, O. B. Knode, and G. C. Callett. This little bi-monthly is well conducted, and we place it on our exchange list with pleasure. The editors will oblige us by sending the first three numbers.

